# Supplementary material for: Acupuncture for Pain Management in Pediatric Patients with Sickle Cell Disease
Source: Children (Basel). 2022 Jul 19;9(7):1076. doi: 10.3390/children9071076 (PMC9324601; doi:10.3390/children9071076)
Supplement: Supplementary file 1 [file children-09-01076-s001.zip › children-1777098-supplementary.pdf]

Table S1. A summary table indicating the most common acupuncture points used for treating acute pain in pediatric patients with SCD from articles in this review.

Acupuncture Point	Studies Included			
	Acupuncture for Sickle Cell Pain Management in Pediatric Emergency Department, Hematology Clinic, and Inpatient Unit by Tsai et al., 2015 [5]	Acupuncture for pain management in children with sickle cell disease By Mahmood, et al., 2020 [6]	Acupuncture as an adjunctive treatment for pain in hospitalized children with sickle cell disease By Reece-Stretman, et al., 2021 [8]	Acupuncture for pediatric sickle cell pain management: A promising non-opioid therapy By Tsai et al, 2020 [9]
Large intestine 11	X			
Large intestine 4	X	X	X	X
Stomach 44	X			
Liver 3	X	X	X	X
Stomach 36	X			X
Spleen 6	X			X
Kidney 3	X			X
Governing Vessel 24.5		X		
Governing Vessel 20		X		
Auricular Shenmen				X
